# Targeting mitochondrial deubiquitinase USP30 to induce mitophagy in heteroplasmic mitochondrial diseases

**DOI:** 10.1007/s43440-026-00829-7

**Published:** 2026-01-26

**Authors:** Brígida R. Pinho, Vasco Martins, Anitta R. Chacko, Célia Nogueira, Michael R. Duchen, Jorge M. A. Oliveira

**Affiliations:** 1 UCIBIO Applied Molecular Biosciences Unit, Mitochondria and Neurobiology Lab, Faculdade de Farmácia, Universidade do Porto, Porto, 4500-313 Portugal; 2 Associate Laboratory i4HB Institute for Health and Bioeconomy, Faculdade de Farmácia, Universidade do Porto, Porto, 4500-313 Portugal; 3 Department of Cell and Developmental Biology, University College London, London, UK; 4 Department of Human Genetics, National Institute of Health Doutor Ricardo Jorge, Porto, Portugal; 5 Departamento de Ciências do Medicamento, Laboratório de Farmacologia, Faculdade de Farmácia, Universidade do Porto, Rua de Jorge Viterbo Ferreira, 228, Porto, 4050-313 Portugal

**Keywords:** Mitochondrion, Mitochondrial DNA, Ubiquitin, Autophagy, ATP synthase, Cybrid

## Abstract

**Background:**

Mitochondrial DNA (mtDNA) diseases are heterogeneous and lack effective treatments. Their severity correlates with mutant mtDNA load. Mitophagy degrades dysfunctional mitochondria, contributing to a healthy mitochondrial pool. USP30, a mitochondrial deubiquitinase, limits mitophagy by removing the ubiquitin tagging mitochondria for degradation. We investigated whether inhibiting USP30 could enhance mitophagy and reduce mutant mtDNA load in a heteroplasmic mitochondrial disease.

**Methods:**

Cybrids cells harboring mutant m.8993T > G mtDNA - common cause of NARP syndrome and maternally inherited Leigh syndrome (MILS) - were treated with USP30 inhibitor MF-094 under glycolytic and oxidative phosphorylation conditions. On-target activity of MF-094 was assessed by mitochondrial ubiquitination (western-blot) and mitolysosome formation (microscopy). The mutation’s effects were investigated on cell proliferation and metabolism (respirometry and ATP levels). The impact of MF-094 on mutant mtDNA load and mtDNA copy number was quantified by PCR.

**Results:**

Comparing with control cells (0% mutant mtDNA), cells with mutant mtDNA exhibited reduced proliferation and ATP levels under oxidative phosphorylation conditions; and reduced oxygen consumption, increased extracellular acidification, and sustained resazurin metabolism after mitochondrial inhibition under glycolytic conditions. MF-094 induced mitophagy via increased mitolysosome formation. Mechanistically, MF-094 showed on-target effects, increasing mitochondrial ubiquitination. However, chronic treatment (3–6 weeks) evoked only a small (5%) non-significant reduction in mutant mtDNA load.

**Conclusions:**

Despite inducing mitophagy, the USP30 inhibitor MF-094 showed little potential to manage m.8993T > G related diseases, as it did not significantly reduce the load of this NARP/MILS causing mtDNA mutation. These results highlight the complexity of mutant mtDNA management and the need for innovative strategies for these disorders.

**Supplementary Information:**

The online version contains supplementary material available at 10.1007/s43440-026-00829-7.

## Introduction

Mitochondrial DNA (mtDNA) diseases are among the most common inherited neurometabolic diseases, often leading to severe disability, shortened lifespan, and significant societal costs [1]. mtDNA differs from nuclear DNA, complicating diagnosis and clinical management of mtDNA diseases: (1) each cell has several mtDNA copies, where mutant mtDNA can coexist with non-mutant mtDNA in a condition called heteroplasmy; (2) mtDNA mutations are functionally recessive, requiring a threshold of mutant mtDNA to impair cellular function and cause symptoms; and (3) individual mtDNA mutations are linked to diverse clinical presentations, leading to variability in the genotype-phenotype correlation [2, 3].

Currently, therapeutic options for mtDNA diseases focus on symptom management through non-pharmacological strategies combined with vitamins and antioxidants [4]. Genetic manipulation is under investigation as a therapeutic option [5–7], but these therapies are still in early stages of development, facing challenges regarding efficiency, off-targets effects, safety and administration routes [8]. This study aims to investigate an alternative or complementary strategy to ongoing research on genetic manipulation, identifying and validating pharmacological targets to modulate mitochondrial quality control and reduce mutant mtDNA load.

Mitochondrial quality control pathways promote mitochondrial fitness and function through diverse pathways, including reactive oxygen species scavenging, protein refolding or degradation, maintenance of mitochondrial fission-fusion balance, biogenesis of new mitochondria, and the degradation of mitochondria by autophagy (mitophagy) [9–11]. Mitophagy is crucial for removing dysfunctional or unnecessary mitochondria and can occur by different pathways. The most studied mitophagy pathway is the PINK1/Parkin-dependent pathway, where mitochondrial damage promotes PINK1 accumulation on the outer mitochondrial membrane (OMM), recruiting the E3 ubiquitin ligase Parkin; Parkin ubiquitinates OMM proteins, which are then recognized by autophagy receptors, triggering autophagic vesicle formation around mitochondria [12, 13].

Promoting autophagy using the general autophagy inducer rapamycin has shown promising results in mitochondrial disease models: rapamycin restored mitophagy in skeletal muscle and partially rescued muscle pathology in a mitochondrial myopathy mouse model [14, 15]; rapamycin also rescued mitochondrial function in cellular models of the mtDNA disease Leber’s hereditary optic neuropathy [16], in human fibroblasts carrying the m.3243 A > G mtDNA mutation [17], and in a mouse model with mtDNA deletions [18]. However, rapamycin broadly induces autophagy by inhibiting mTORC1, a protein kinase that regulates multiple other processes, including metabolism and immune responses [19]. Consequently, rapamycin effects are not specific to mitochondria and can broadly impact cell biology. Therefore, identifying new, specific mitophagy inducers with low toxicity is essential for developing targeted pharmacological strategies to treat mitochondrial diseases [20].

Ubiquitination is reversed by deubiquitinase enzymes, which remove ubiquitin from proteins tagged for degradation [21]. USP30 is a transmembrane deubiquitinase localized at the OMM and peroxisomes [22]. At mitochondria, USP30 regulates protein import [23], fission-fusion dynamics [24], and the ubiquitination status of key OMM proteins, thereby preventing inadvertent mitochondrial engagement by the autophagy machinery [22]. Inhibition of USP30 activity, either pharmacologically or via genetic silencing, has been shown to induce mitophagy and demonstrate therapeutic potential in Parkinson’s disease models [25–28]. The therapeutic potential of targeting USP30 has also been investigated in Alzheimer’s disease [29], traumatic brain injury [30], subarachnoid hemorrhage [31] myocardial cell senescence [32], and very recently in context of mitochondrial diseases [33], with promising results in attenuating disease phenotypes; however, to our knowledge, it remains to be investigated in m.8993T > G mutation related-disorders, such as Maternally Inherited Leigh Syndrome (MILS) and Neuropathy, Ataxia, Retinitis Pigmentosa syndrome (NARP). Here, we investigated USP30 inhibition as a potential new experimental strategy for mitochondrial diseases, studying whether USP30 inhibition could induce mitophagy and reduce the mutant mtDNA load in a heteroplasmic cell model.

## Materials and methods

### Cybrid cell lines

The 143B cybrid cell lines with approximately 0% (N0), 40% (N40) and 80% (N80) of the m.8993T > G mtDNA mutant loads were a gift from the Duchen Lab [17], originated in the Minczuk Lab [34]. Cells were maintained in DMEM (Gibco, 31966021) supplemented with 10% fetal bovine serum (FBS, Gibco, A5256801) and 1% penicillin/streptomycin (Gibco, 15140122), at 37 °C, in a humidified atmosphere with 5% CO_2_ [17]. When conditions of oxidative phosphorylation (OXPHOS) dependence were required, cells were cultured in galactose medium (without glucose) as we previously described [35].

### Cell treatments

Cybrids were treated with the USP30 inhibitor MF-094 (Sigma-Aldrich, SML2501) acutely (2–24 h) or chronically (3 or 6 weeks in OXPHOS-dependent or glycolytic conditions, respectively). Previous work showed that a treatment period of 6 weeks was required to detect a change in mutant mtDNA load in a cybrid model of a heteroplasmic mitochondrial disease in glycolytic conditions [17]. A stock solution of MF-094 was prepared in DMSO anhydrous (Sigma-Aldrich, 276855) and the solvent concentration was kept constant across all conditions in each experiment.

For chronic treatments, cells were seeded in 6-well plates at a density of 5,000–12,500 cells/cm^2^ and passaged every 5–7 days: cells were trypsinised with 0.25% trypsin-EDTA (Gibco, 25200072) for 5 min and counted using a Corning Cyto Smart Cell Counter to determinate the cell density. After passaging, the remaining cells were pelleted and frozen at -80 °C for subsequent DNA extraction. Importantly, medium with solvent or MF-094 was renewed every 2–3 days. Doubling time was calculated using the formula: doubling time = period of time between cell seeding and counting × ln (2) / ln (number of cells at the end / number of cells at the start) [36].

### MF-094 quantification

MF-094 was quantified by spectrophotometry [37], with absorbance measured in the 200–400 nm range (2 nm steps) using a BioTek Synergy HT microplate reader and 96-well UV-transparent plates (Greiner, 655801). The absorbance spectrum and calibration curve were performed using increasing concentrations of MF-094 diluted in glucose-containing cell culture medium described in subsection “Cybrid cell lines”. Background absorbance from the medium (medium with DMSO and without MF-094) was subtracted from the absorbance of the MF-094 solutions. To assess MF-094 levels over time under cell culture conditions, cells were seeded on 24-well plates at a density of 2,500 cells/cm^2^ and treated with 30 µM of MF-094, a non-toxic concentration that provides a suitable absorbance-to-noise ratio for drug quantification. All conditions - cells treated with MF-094, or treated only with solvent, and the respective conditions without cells – were incubated at 37 °C in a humidified atmosphere with 5% CO_2_ for 24, 48 and 72 h. Supernatant from each condition was transferred to a 96-well plate for absorbance analysis as described at the beginning of this section.

### Cell proliferation

Cell proliferation was assessed under conditions that support glycolytic vs. OXPHOS-metabolism (glucose medium vs. galactose medium) by brightfield live microscopy over time. Cells were seeded in 96-well plates at a density of 12,000 cells/cm^2^ and imaged at 4, 24, 48 and 72 h after seeding using a 10x objective on an inverted fluorescence microscope (Eclipse TE300, Nikon) equipped with an ORCA-ER camera (Hamamatsu). Cell counts were performed using ImageJ by a researcher blinded to experimental conditions.

### Resazurin metabolism

Resazurin metabolism was evaluated under glycolytic vs. OXPHOS-dependent conditions. Cells were seeded in 96-well plates at a density of 60,000 cells/cm^2^. After 48 h of seeding, cells were treated with increasing concentrations of MF-094 or mitochondrial inhibitors: rotenone (Sigma-Aldrich, R8875), myxothiazol (Sigma-Aldrich, T5580), antimycin (Sigma-Aldrich, A8674) and oligomycin (Sigma-Aldrich, O-4876). After 20 h of treatment, 40 µM resazurin (Sigma-Aldrich, R7017) was added to the cells and the reduction of resazurin into resorufin was monitored as we previously described [35]. After measuring resorufin fluorescence, the supernatant was carefully discarded, and cells were incubated for 30 min with 1 µg/mL Hoechst 34580 (Sigma-Aldrich, 63493) to estimate cell density. Following washing with phosphate-buffered saline (PBS), Hoechst fluorescence was assessed by 530 nm excitation and 590 nm emission in a BioTek Synergy HT microplate reader.

### ATP quantification

ATP and ADP levels were quantified by HPLC in cell lysates, as previously described [38, 39] with some modifications. Cells seeded in 6-well plates at a density of 8,000 cells/cm^2^ were grown to near-confluence and washed twice with PBS. After adding 80 µL of 0.3 M perchloric acid (Merck, C836618), the plates were cooled for 10 min at -80 °C and then thawed on ice. Cells were scraped, transferred into microfuge tubes, and centrifuged at 3,000 × *g* (10 min, 4 °C). The pellet was stored at -80 °C for subsequent protein quantification. The pH neutralization and clearance of the supernatant, along with ATP and ADP quantification, were performed as described in [39]. ATP (Sigma-Aldrich, A7699) and ADP (Sigma-Aldrich, A2754) standards were used to create calibration curves. ATP levels were normalized by ADP levels or by the protein content of the sample protein, determined using the Bradford assay (Bio-Rad, 500-0006).

### Oxygen consumption and extracellular acidification rates

Measurements of oxygen consumption rate (indicative of aerobic respiration) and extracellular acidification (indicative of glycolysis) were performed using the Seahorse XF24 Extracellular Flux Analyser (Seahorse Biosciences) [17]. Cells seeded in XF24 cell culture plates (Agilent) at a density of 70,000 cells/cm^2^ were treated with MF-094 or solvent for 24 h. On the day of the assay, cells were washed twice with PBS and equilibrated in the experimental medium [DMEM (Sigma-Aldrich, D5030); 2 mM GlutaMax (Gibco, 35050038); 1 mM sodium pyruvate (Sigma-Aldrich, P8574)] at 37 °C in a CO_2_-free incubator for 45 min to 1 h prior to the assay. Immediately before the assay, the medium was replaced with fresh experimental medium at 37 °C. After measuring basal respiration, compounds/drugs were added sequentially to each well in the following order: 25 mM glucose (Sigma-Aldrich, 49159), 0.5 µM oligomycin (Focus Biomolecules, 10-2092), 2 µM FCCP (TargetMol, T6834), and a mixture of 1 µM rotenone with 1 µM antimycin. In control experiments, 50 mM 2-deoxy-D-glucose (Sigma-Aldrich, D6134) was added after glucose and oligomycin as a control to abolish glycolytic activity. Data were analysed using Seahorse Analytics (Agilent). Oxygen consumption and extracellular acidification rates were normalized to the baseline control (without cells) and to the protein content of each well, which was quantified post-assay using the Bradford assay.

### Acidification of culture medium

Culture medium acidification was measured through ratiometric absorbance analysis of the pH indicator phenol red. Cybrid cells were cultured in T-25 flasks or 6-well plates (initial cell density 8,000 cells/cm^2^) in glucose-containing medium with phenol red (hereafter referred to as glucose medium). At a near-confluent cell density, 200 µL of supernatant culture medium from each condition was transferred into a 96-well plate and the phenol red absorbance measured as previously described [17]. Cells were then trypsinised and counted to calculate the cell density. Results were expressed as the absorbance 443/570 ratio corrected for cell density.

### Mutant mtDNA load quantification

Mutant mtDNA load was assessed by Allele Refractory Mutation System (ARMS)-based quantitative PCR (qPCR) analysis, which allows the quantification of mtDNA point mutations by measuring the relative amounts of wild-type and mutant mtDNA in a single step [40]. DNA was extracted from frozen cell pellets using the E.Z.N.A.^®^ Tissue DNA Kit (Omega Bio-Tek, D3396-02) following the manufacturers’ instructions, and quantified using the Microplate Take3 system in a BioTek Synergy HT microplate reader. qPCR amplification was performed as previously described [17] using SsoAdvanced Universal SYBR Green Supermix (Bio-Rad, 1725271) and primers detailed in [40]. Mutant heteroplasmy level (%) was calculated using the following formula 1/[1 + (1/2) ^ΔCT^] × 100%, where ΔCT = CT_wild−type_ – CT_mutant_ [40].

### mtDNA copy number

The relative mtDNA copy number was determined using qPCR with primers for the mtDNA tRNA^Leu(UUR)^ and for the nuclear b2-microglobulin [41]. DNA extraction and quantification were performed as described in the Sect. “Mutant mtDNA load quantification”. qPCR was performed using SsoAdvanced Universal SYBR Green Supermix, following the protocol previously described [17]. The relative mtDNA content was calculated using the following formula: 2 × 2 ^ΔCT^, where ΔCT = CT_nuclearDNA_^–^ CT_mtDNA_ [41].

### Mitochondrial ubiquitination

We performed a mitochondria-enriched protein extraction as we previously described, with some modifications [39]. Cells cultured in 6-well-plates at an initial density of 8,000 cells/cm^2^ were treated with MF-094 or solvent for 2 h. After washing with ice-cold PBS, cells were scraped into sucrose lysis buffer [39], sonicated, and subjected to 3 freeze-thaw cycles. The lysates were centrifuged at 1,200 × *g* (5 min, 4 °C) to pellet cell debris and nuclei, followed by centrifugation at 16,600 × *g* (25 min, 4 °C) to isolate mitochondria-enriched pellets, which were then resuspended in ice-cold RIPA buffer [42]. Protein concentration was quantified using the Bradford protein assay.

Protein extracts were denatured [42], loaded (30 µg) onto either gradient 4–12% pre-cast gels (Invitrogen, NW04120BOX) or hand-made 10% polyacrylamide gels and electrophoresed for 35 min at 200 V, or 90 min at 120 min, respectively. The resolved proteins were then transferred to PVDF membranes (Millipore, IPVH00010) overnight at 30 V (4 °C). Membranes were blocked overnight in 5% bovine serum albumin (NzyTech, MB04602) prepared in PBS with 0.05% Tween 20 (Sigma-Aldrich, P9416), and then incubated with primary and respective horseradish peroxidase-conjugated secondary antibodies. Primary antibodies: anti-ubiquitin produced in mouse (Santa Cruz Biotechnology, sc-8017, RRID AB_628423–1:500, overnight); anti-TOM20 produced in rabbit (Proteintech, 11802-1-AP, RRID: AB_2207530–1:1000, 1 h). Secondary antibodies: anti-mouse (Invitrogen, G-21040, RRID AB_2536527–1:4000, 1 h); anti-rabbit (Invitrogen, G-21234, RRID AB_2536530–1:4000, 1 h). Detection was achieved using the Novex ECL Chemiluminescent kit (Invitrogen, WP20005) and a ChemiDoc MP Imaging system (Bio-Rad). Membranes were stained with Coomassie (Sigma-Aldrich, 27816) for protein loading control.

### Mitolysosome quantification

Mitolysosome numbers were quantified by imaging of cells transfected with the mitochondrial protein COX8 tagged with EGFP and mCherry. Cells were seeded in glass-bottom Ibidi chamber slides (Ibidi, 80827) at a density of 15,000 cells/cm^2^, washed with DMEM (Gibco, 31966021), and incubated with a mixture of Lipofectamine LTX (Invitrogen, 15338030), Reagent plus and plasmid DNA (0.5 µg pCLBW-cox8-EGFP-mCherry, Addgene, 78520 [43]) at a ratio of 2:1:1. After 45 min, cells were washed again, and the usual culture medium was restored. 16 h post-transfection, the medium was replaced with imaging medium (DMEM (Gibco, A1443001) supplemented with 25 mM glucose, 1 mM sodium pyruvate, 4 mM GlutaMax, 10% FBS, 1% penicillin/streptomycin and 5 mM HEPES), and cells were treated with MF-094 or solvent control. Imaging was performed 24 h after treatment using the aforementioned microscope with a monochromator (Polychrome II, Photonics), exciting EGFP at 488 nm and mCherry at 575 nm, with emissions collected using band-pass filters (Chroma). Image acquisition used identical, non-saturating equipment settings for intensity comparisons. Mitolysosome quantification was performed as previously described [44] and expressed as the number of mitolysosomes per cell and their size.

### Data and statistical analysis

Graphical representations and statistical analysis were performed using Prism 9.0 (GraphPad Software). Non-linear regressions of cell proliferation were performed using an exponential growth equation and Extra sum-of-squares F test was used to assess if one curve adequately fits all the data sets, providing information on the similarity between data sets. Resazurin metabolism data were fitted to a sigmoidal non-linear curve. A simple linear regression was used to determine the equation of the calibration curve of MF-094 quantification. The Shapiro-Wilk normality test was used to assess data distributions. For normally distributed data: values are presented as mean ± standard error of the mean (SEM) from *n* experiments specified in figure legends; single-factor analyses were performed using one-way ANOVA with Dunnett’s multiple comparisons test (vs. control); multifactorial analyses (two-way ANOVA) were used to evaluate interactions and main effects between the factors genotype and drug concentration. When genotype or drug concentration effects were significant, Dunnett’s multiple comparisons test was applied to identify differences between mutant cells (N40 and N80) and wild-type cells (N0) or between MF-094-treated conditions and control (solvent-treated). For non-normally distributed data: values are presented as median and interquartile range, with extremes presenting 10–90 percentiles; the Mann-Whitney test was used to compare two groups. For all analyses, a *p* value under 0.05 was considered statistically significant.

## Results

### Cells harboring the m.8993T > G mutation exhibit reduced mitochondrial respiratory activity and high reliance on glycolysis

**Fig. 1:**
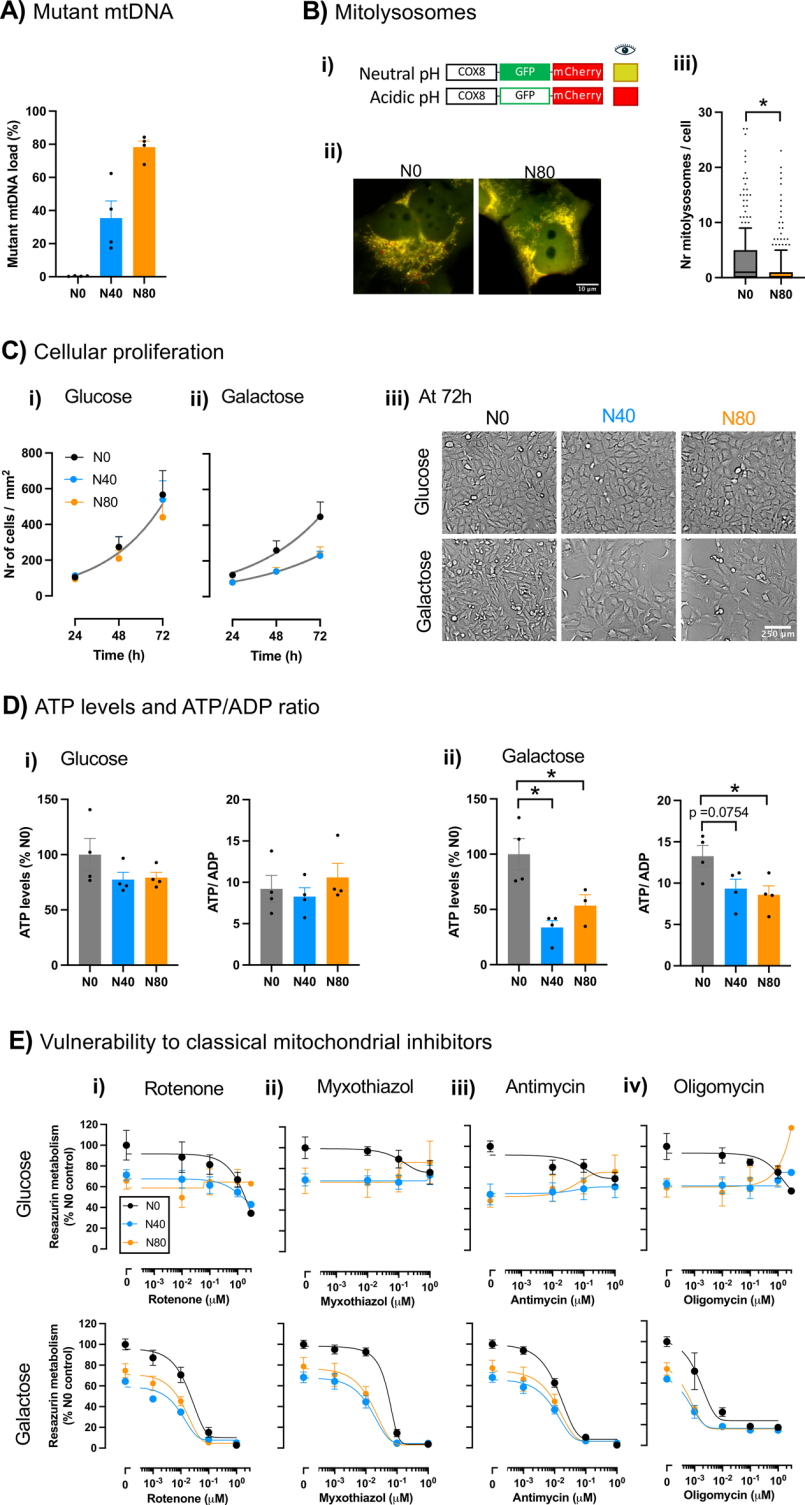
Mutant cells exhibit reduced proliferation and ATP levels under OXPHOS-dependent conditions and resistance to mitochondrial inhibition under glycolytic conditions. 143B cybrid cells harboring 0% (N0), 40% (N40) or 80% (N80) of the m.8993T > G mtDNA mutation were cultured under glycolytic (glucose-containing medium) or OXPHOS-dependent conditions (galactose-containing medium – without glucose). **(A)** Confirmation of the mutant mtDNA load by ARMS-PCR. Data are mean ± SEM, *n* = 4 independent experiments **(B)** Mitophagy of N0 and N80 cells under glycolytic conditions was assessed by live imaging of the mitochondrial protein COX8 linked to both EGFP and mCherry: **(i)** schematic representation of the dual-fluorescent mitochondrial protein COX8-EGFP-mCherry: at neutral/alkaline pH, both EGFP and mCherry fluoresce, while at acidic pH (e.g. in mitolysosomes), only mCherry fluorescence is retained; **(ii)** representative images – red dots identify mitolysosomes (mCherry fluorescence only); **(iii)** quantification of the number of mitolysosomes per cell. *n* = 312–318 cells, from 3 independent experiments. Results are presented as median and interquartile range, with extremes presenting 10–90 percentiles, *p < 0.05, Mann-Whitney test. **C)** Cellular proliferation: growth over time after seeding in **(i)** glucose- or **(ii)** galactose-containing medium; **(iii)** representative images at 72 h after seeding. Initial cell density was similar across all conditions. Data are mean ± SEM, *n* = 5–7 independent experiments, non-linear regression following exponential growth equation. We used extra sum-of-squares F test for statistical comparison of growth curves: a single curve fits all data under glycolytic conditions (glucose), but not in OXPHOS-dependent conditions (galactose). **D)** ATP levels and ATP/ADP ratio quantified by HPLC of cells cultured in **(i)** glucose- and **(ii)** galactose-containing medium. Data are mean ± SEM, *n* = 3–4 independent experiments; *p < 0.05, one-way ANOVA followed by Dunnett’s multiple comparisons test. **E)** Resazurin metabolism after treatment of cells cultured in glucose- and galactose-containing medium with increasing concentrations of classical mitochondrial inhibitors for 24 h: **(i)** rotenone (complex I inhibitor); **(ii)** myxothiazol (complex III inhibitor); **(iii)** antimycin (complex III inhibitor); **(iv)** oligomycin (ATP synthase inhibitor). Data are mean ± SEM in % of N0 without drug treatment; *n* = 4 independent experiments

To characterize the cell model of mtDNA disease, we compared 143B cybrid cells harboring approximately 0, 40 and 80% of the m.8993T > G mtDNA mutation (Fig. 1A). Initially, we studied the impact of the m.8993T > G mtDNA mutation on mitophagy by assessing the number of mitolysosomes in wild-type cells (N0) and cells harboring 80% of the mutation (N80) under glycolytic conditions. Mutant cells showed a lower number of mitolysosomes (Fig. 1B), suggesting decreased mitophagy (U = 35278, *n*_N0_= 312, *n*_N80_= 318). Then, we measured cellular proliferation rates and metabolism in glycolytic- (medium with glucose) and OXPHOS-dependent conditions, in which glucose was replaced by galactose.

Under glycolytic conditions, cells with mutant mtDNA (mutant cells; N40, N80) proliferated at a similar rate to wild-type cells (N0) (Fig. 1C*i*). In contrast, under OXPHOS-dependent conditions mutant cells grew more slowly than wild-type cells (Fig. 1C*ii*, *iii*), suggesting a bioenergetic impairment in mutant cells that limits the cell cycle. To assess such bioenergetic impairment, we quantified the cellular ATP levels. Under glycolytic conditions, mutant and wild-type cells exhibited similar ATP levels (*F*_2,9_= 1.683) and ATP/ADP ratio (*F*_2,9_= 0.6038) (Fig. 1D*i*). In contrast, under OXPHOS-dependent conditions, mutant cells showed significantly reduced ATP levels (*F*_2,8_= 10.72) compared to wild-type cells (Fig. 1D*ii*). Additionally, mutant cells harboring 80% of mutation also showed reduced ATP/ADP ratio (*F*_2,9_= 4.534), supporting the impairment of mitochondrial ATP production.

We next challenged cells with classical mitochondrial inhibitors – rotenone, myxothiazol, antimycin and oligomycin. Exposure to these inhibitors reduced resazurin metabolism in wild-type cells in a concentration-dependent manner under both glycolytic and OXPHOS-dependent conditions (Fig. 1E). As expected, the reduction in resazurin metabolism of wild-type cells treated with mitochondrial inhibitors was more pronounced under OXPHOS-dependent conditions, where cells are forced to use the respiratory chain to be bioenergetically efficient compared to glycolytic conditions. Interestingly, for mutant cells under glycolytic conditions, exposure to classical mitochondrial inhibitors either maintained or increased their resazurin metabolism (excepting rotenone in cells harboring 40% of mutation – Fig. 1E*i*). Under OXPHOS-dependent conditions, exposure to classical mitochondrial inhibitors reduced resazurin metabolism in mutant cells to values close to zero at higher concentrations. These results from resazurin metabolism are proportional to the cell density measured by DNA staining with Hoechst 34580 (Supplementary Fig. 1), indicating the susceptibility to mitochondrial inhibitors that either hinder cell proliferation or induce cell death under glycolytic or OXPHOS-dependent conditions in these cells. Notably, the increase in resazurin metabolism in cells harboring 80% mutant mtDNA treated with the highest concentration of antimycin (Fig. 1E*iii*) or oligomycin (Fig. 1E*iv*) is not accompanied by a corresponding increase in cell density (Supplementary Fig. 1), suggesting a state of mitochondrial inactivity characterized by elevated NADH/NADPH levels, which could drive increased resazurin reduction via dehydrogenases [45]. Additionally, untreated mutant cells under OXPHOS-dependent conditions exhibited reduced resazurin metabolism compared to untreated wild-type cells (Fig. 1E), yet this reduction did not correlate with a decrease in cell density (Supplementary Fig. 1), highlighting the lower metabolism of mutant cells in the absence of glucose. Overall, the decreased susceptibility of mutant cells to mitochondrial inhibitors under glycolytic conditions suggests that their respiratory chain activity is reduced, producing energy mainly by glycolysis.

**Fig. 2:**
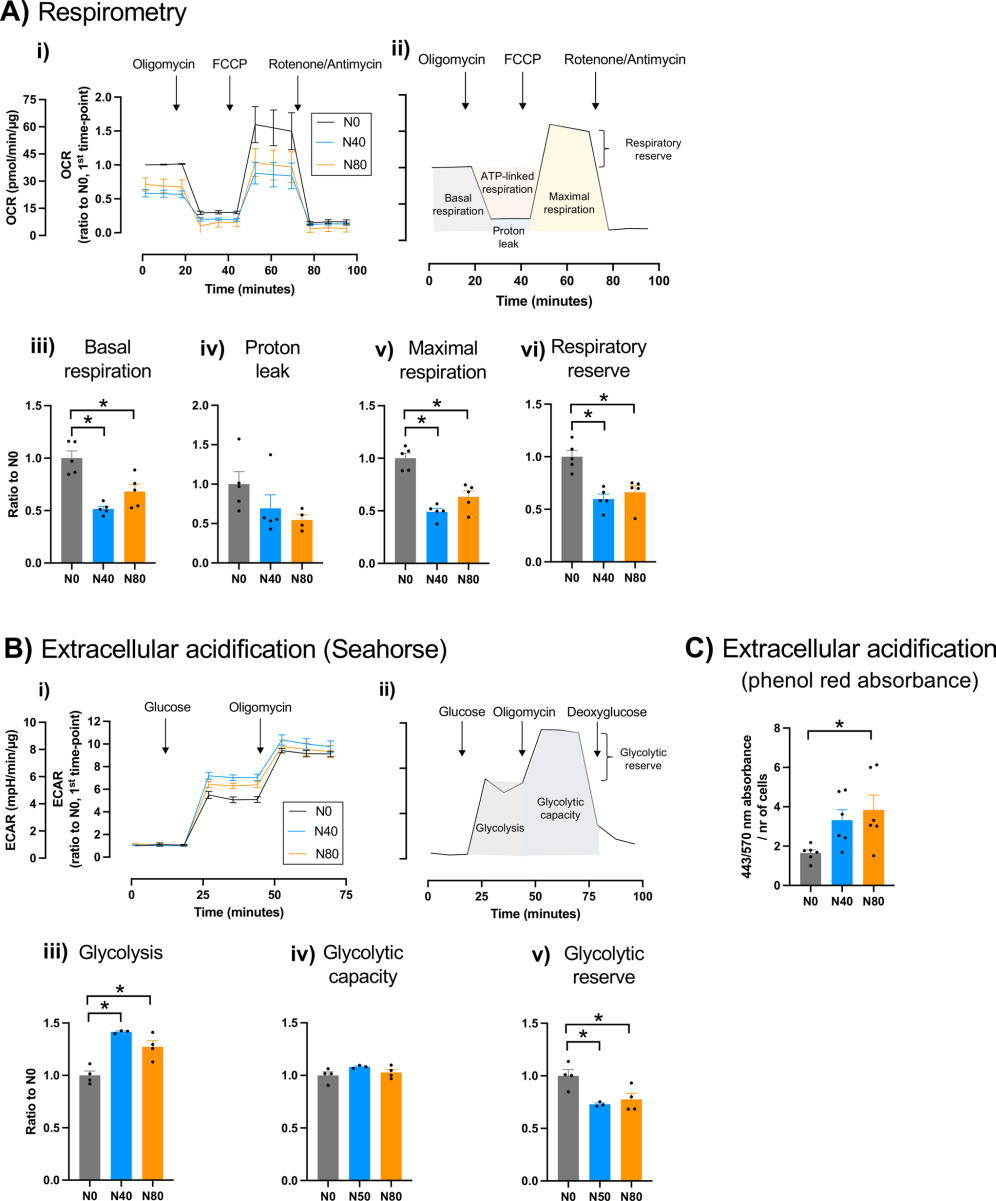
Mutant cells have reduced mitochondrial respiration and increased extracellular acidification compared to wild-type cells. **A)** Oxygen consumption rates (OCR) of wild-type (N0) and mutant (N40 and N80) cells were assessed under glycolytic conditions and using a Seahorse XF24 Extracellular Flux Analyser. Cells were sequentially challenged with 0.5 µM oligomycin, 2 µM FCCP and 1µM rotenone + 1 µM antimycin: **(i)** OCR profiles; **(ii)** schematical representation of an OCR profile, highlighting basal respiration, ATP-linked respiration, maximal respiration, proton leak and respiratory reserve; quantification of **(iii)** basal respiration, **(iv)** proton leak, **(v)** maximal respiration and **(vi)** respiratory reserve. *n* = 5 independent experiments. **B)** Extracellular acidification rate (ECAR) assessed using a Seahorse XF24 Extracellular Flux Analyser: **(i)** ECAR profile of cells in response to 25 mM glucose followed by 0.5 µM oligomycin; **(ii)** ECAR profile of cells that were treated with 50 mM 2-deoxy-D-glucose after glucose and oligomycin to abolish glycolysis; this graphical representation identifies the parameters: glycolysis, glycolytic capacity and glycolytic reserve; quantification of **(iii)** glycolysis rate, **(iv)** glycolytic capacity, and **(v)** glycolytic reserve. *n* = 4 independent experiments. **C)** Extracellular acidification induced by cells quantified by the ratiometric absorbance of phenol red at 443 nm / 570 nm ratio, normalized by cell count. *n* = 6 independent experiments. **Aiii - Avi**,** Biii - Bv**,** C)** Data are mean ± SEM, *p < 0.05, one-way ANOVA with Dunnett’s multiple comparisons test

We confirmed the reduced activity of the respiratory chain and the increased dependence on glycolysis of mutant cells through respirometry and analysis of extracellular acidification. Under glycolytic conditions, oxygen consumption rate was reduced in the mutant cells (Fig. 2A), with a decrease in basal respiration (*F*_2,12_= 18.53), maximal respiratory capacity (*F*_2,12_= 31.70) and respiratory reserve (*F*_2,12_= 14.35), compared to wild-type cells. Moreover, in the Extracellular Acidification Rate (ECAR) assay (Seahorse, Agilent), mutant cells showed increased rate of extracellular acidification after glucose addition (Fig. 2B*i*, *iii*; further confirmed by the phenol red assay in (*F*_2,15_= 4.478) Fig. 2C), indicating a higher glycolysis rate compared to wild-type cells (*F*_2,8_= 20.56). However, mutant and wild-type cells exhibited similar maximal glycolytic capacity (*F*_2,8_= 1.922) (Fig. 2B*i*, *iv*), and thus mutant cells have a decreased glycolytic reserve (*F*_2,8_= 7.348) (Fig. 2B*v*).

In summary, these results show that cells harboring the m.8993T > G mutation present a disease phenotype characterized by reduced mitochondrial activity, being a well-suited model for studying potential therapeutic strategies aimed at reducing mutant mtDNA load and attenuating the associated disease phenotype.

### Pharmacological inhibition of USP30 induces mitochondrial ubiquitination and mitolysosome formation

**Fig. 3:**
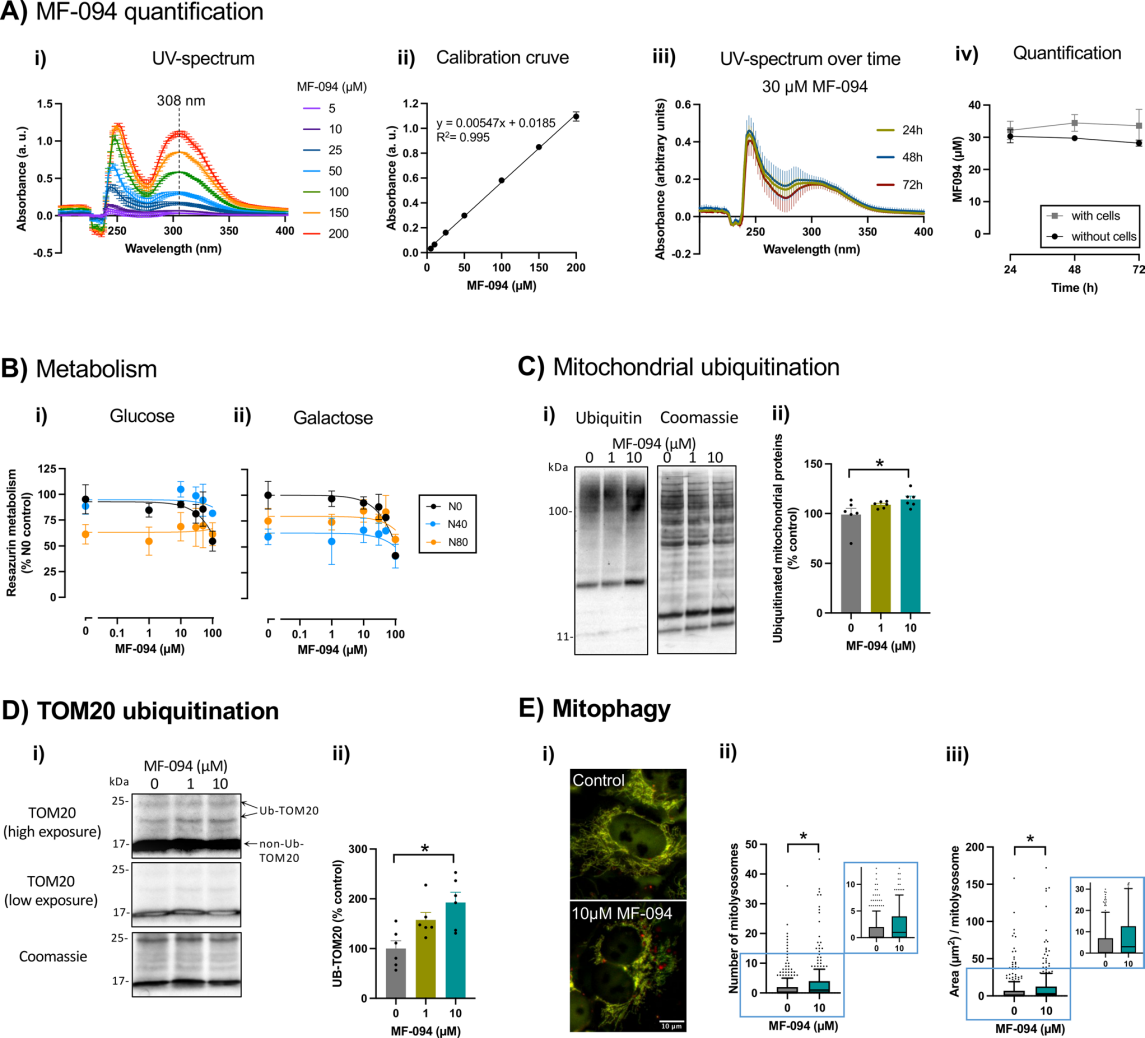
MF-094 promotes mitochondrial protein ubiquitination and formation of mitolysosomes. **A)** Quantification of MF-094 by UV-spectrophotometry: **(i)** UV spectrum of increasing concentrations of MF-094, showing an absorbance peak at 308 nm; **(ii)** calibration curve of MF-094: absorbance at 308 nm as a function of concentration; **(iii)** UV spectrum of 30 µM MF-094 after 24, 48, and 72 h of incubation under cell culture conditions in presence of cells; **(iv)** quantification of MF-094 in cell culture medium, with and without cells (initial concentration: 30 µM). Results from 3 independent experiments are presented as mean ± SEM. **B)** Metabolism of resazurin in cells treated with increasing concentrations of MF-094 under **(i)** glycolytic (glucose) or **(ii)** OXPHOS-dependent conditions (galactose); results from 3 to 4 independent experiments are expressed as mean ± SEM, as percentage of N0 cells without drug treatment. **C)** Mitochondrial protein ubiquitination was assessed by western-blot using an antibody against ubiquitin and a mitochondria-enriched extract from N80 cells treated with increasing concentrations of MF-094 under glycolytic conditions for 2 h: **(i)** representative blot and **(ii)** respective quantification. *n* = 6 independent experiments. **D)** TOM20 ubiquitination after N80 cell treatment with increasing concentrations of MF-094 under glycolytic conditions for 2 h: **(i)** representative blot – the panel identified with “high exposure” highlights ubiquitinated TOM20 (higher molecular weight than non-ubiquitinated TOM20); **(ii)** ubiquitinated TOM20 quantification. *n* = 6 independent experiments. **C**,** D)** Results are presented as mean ± SEM, *p < 0.05, one-way ANOVA with Dunnett’s multiple comparisons test. **E)** Mitophagy of N80 cells treated with 10 µM MF-094 under glycolytic conditions for 24 h was assessed by live imaging of the mitochondrial protein COX8 linked to both EGFP and mCherry: **(i)** representative images – red dots identify mitolysosomes (mCherry fluorescence only); quantification of the **(ii)** number and **(iii)** average area of mitolysosomes per cell. The inset graphs show amplified images of the regions of interest in the main graphs. *n* = 349–385 cells, from 4 independent experiments. Results are presented as median and interquartile range, with extremes presenting 10–90 percentiles, *p < 0.05, Mann-Whitney test

Changes in mutant mtDNA load can be a slow process, as demonstrated in a study where rapamycin required at least six weeks of treatment to reduce the mutant mtDNA load in cybrid cells harboring the m.3243 A > G MELAS mutation [17]. To perform a similar chronic treatment with the USP30 inhibitor MF-094 lasting several weeks, we first studied its stability under cell culture conditions. MF-094 exhibits an absorbance peak at 308 nm that changes linearly with concentrations from 5 to 200 µM (Fig. 3A*i*,* ii*). By reading MF-094 absorbance spectra in cell culture medium after exposure to cells over time, we found that MF-094 absorbance remained stable for at least 72 h (two-way ANOVA analysis: interaction, *F*_2,12_= 0.2290; factor time, *F*_2,12_= 0.09722; factor cell presence, *F*_1,12_= 3,150) (Fig. 3A*iii*,* iv*). Thus, we decided to renew the culture media with solvent or MF-094 every 2–3 days (48–72 h) during the cellular chronic treatment.

Next, we evaluated the effects of MF-094 on the resazurin metabolism of wild-type and mutant cells under glycolytic and OXPHOS-dependent conditions and we observed a trend toward decreased metabolism at concentrations of 30 µM or higher (Fig. 3B). Previous studies report that MF-094 induces mitophagy at concentrations above 0.5 µM in primary neurons and C2C12 myotubes [31, 46]. Based on this previous activity data (above 0.5 µM) and on our resazurin metabolism results (no evidence of toxicity up to 30 µM), we selected the concentrations of 1 and 10 µM MF-094 to investigate its on-target activity. Given that the MF-094 target is the USP30 mitochondrial deubiquitinase, we evaluated the overall ubiquitination of mitochondria-enriched extracts from N80 cells via western-blot, as well as the levels of ubiquitinated TOM20, a known USP30 target [25]. Levels of ubiquitinated mitochondrial proteins (*F*_2,15_= 3.522) and of TOM20 (*F*_2,15_= 7.353) were significantly increased in cells treated with 10 µM MF-094 (Fig. 3C, D). To investigate whether this increase in mitochondrial ubiquitination triggers mitophagy, we quantified the number and size of mitolysosomes in treated and untreated N80 cells. Treatment with MF-094 significantly increased the number (U = 55614, *n*_0µM_= 385, *n*_10µM_= 349) and the size (U = 56120, *n*_0µM_= 385, *n*_10µM_= 349) of mitolysosomes (Fig. 3E).

Having characterized the disease phenotypes of the cell model and confirmed the MF-094 on-target activity and its efficacy as mitophagy inducer, we next investigated whether treatment with MF-094 altered the cellular disease phenotypes and, specifically, whether it reduced the mutant mtDNA load.

### Pharmacological USP30 inhibition changes cell proliferation rate, but did not alter mutant mtDNA load

**Fig. 4:**
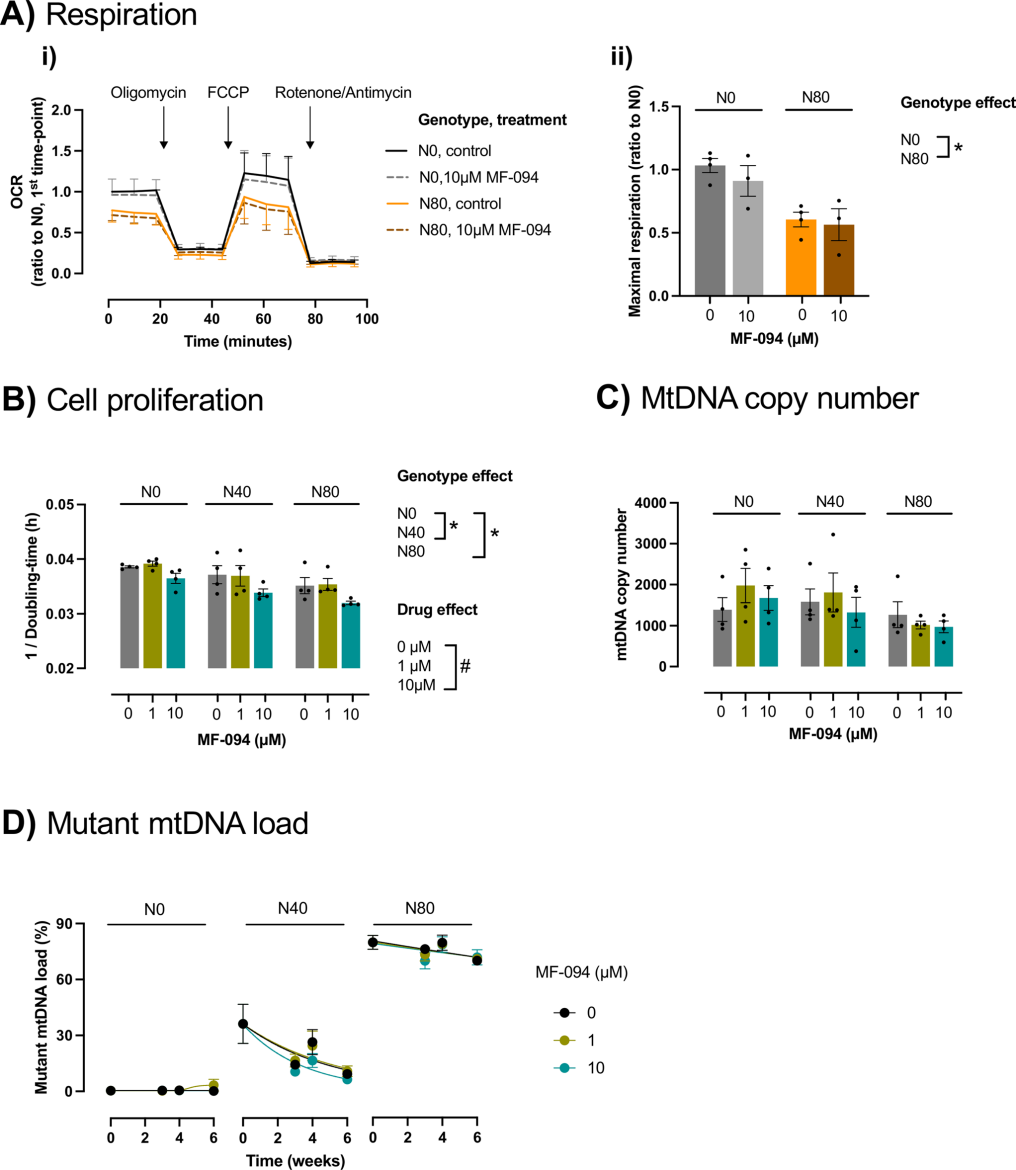
MF-094 induces changes in cellular proliferation, but it did not change mutant mtDNA load. **A)** Oxygen consumption rate (OCR) of cells treated with solvent control or 10 µM MF-094 for 24 h were measured using a Seahorse XF24 Extracellular Flux Analyser. Cells were sequentially challenged with 0.5 µM oligomycin, 2 µM FCCP and 1µM rotenone + 1 µM antimycin: **(i)** OCR profiles; **(ii)** maximal respiration. Results from 3 to 4 independent experiments are expressed as mean ± SEM, two-way ANOVA: *p < 0.05, genotype effect. **B-D)** Cells were treated with increasing concentrations of MF-094 over 6 weeks under glycolytic conditions. **B)** Cell proliferation after 6 weeks of treatment expressed in 1 / doubling time (h). **C)** mtDNA copy number quantified by PCR after 6 weeks of treatment. **D)** Mutant mtDNA load over time. **B-D)** Results are expressed as mean ± SEM from 4 independent experiments, two-way ANOVA followed by Dunnett’s multiple comparisons test to identify differences between genotypes: *p < 0.05, genotype effect; ^#^*p* < 0.05, drug effect

First, we assessed whether a short 24 h treatment with 10 µM MF-094 sufficed to impact the mitochondrial oxygen consumption differences of mutant (N80) vs. control (N0) cells. We found that the significant differences in maximal respiration of N80 vs. N0 cells persisted in the presence of this short 24 h treatment with MF-094 (two-way ANOVA interaction *F*_1,10_= 0.2123; genotype *F*_1,10_= 10.59; treatment *F*_1,10_= 0.8682) (Fig. 4A). To determine whether USP30 inhibition reduces mutant mtDNA load, we treated cells with MF-094 over six weeks under glycolytic conditions. Weekly assessments included cell proliferation and mutant mtDNA load quantification. MF-094 (10 µM) treatment delayed cell proliferation in both mutant and wild-type cells after 6 weeks of treatment (decreased 1/doubling time vs. solvent-treated cells; two-way ANOVA: interaction *F*_4,27_= 0.1708; genotype *F*_2,27_= 10.29; treatment *F*_2,27_= 7.645) (Fig. 4B). Given that MF-094 induced mitophagy (Fig. 3E), we assessed whether it altered mtDNA copy number and mutant mtDNA load. mtDNA copy number tended to be lower in cells harboring 80% of mutant mtDNA compared to wild-type cells (two-way ANOVA: interaction F_4,27_= 0.5289; genotype F_2,27_= 2.926 (*p* = 0.0708); treatment F_2,27_= 0.5870) (Fig. 4C). Treatment with MF-094 did not significantly alter mtDNA copy number (interaction *F*_4,27_= 0.5289; genotype *F*_2,27_= 2.926; treatment *F*_2,27_= 0.5870) (Fig. 4C), or relative mutant mtDNA load (Fig. 4D). These results suggest that under the glycolytic conditions, MF-094 stimulates mitophagy and slows cellular proliferation, but is unable to decrease mutant mtDNA load.

**Fig. 5:**
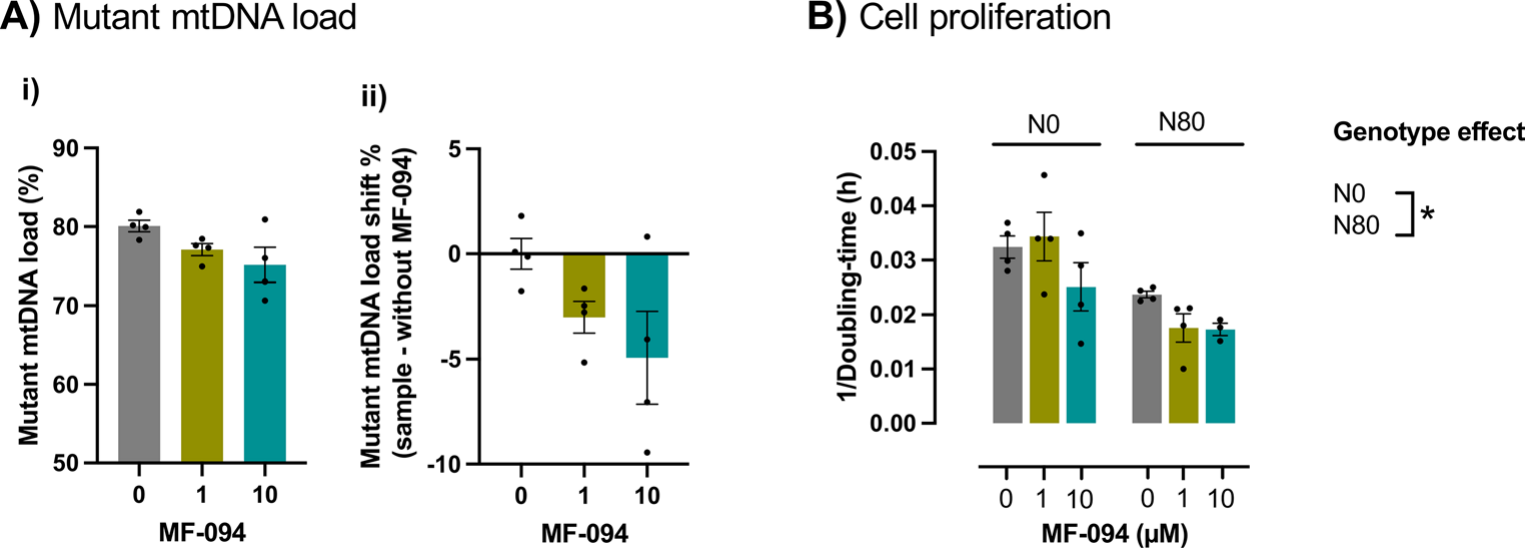
MF-094 did not significantly reduce mutant mtDNA load under OXPHOS-dependent conditions. Cells harboring 80% mutant mtDNA load were treated with increasing concentrations of MF-094 for 3 weeks under OXPHOS-dependent conditions. **A)** Mutant mtDNA load after 3 weeks of treatment: **(i)** % of mutant mtDNA load; **(ii)** change in mutant mtDNA load relative to cells treated with solvent (without MF-094). **B)** Cell proliferation after 3 weeks of treatment expressed in 1 / doubling time (h). **A**,** B)** Results are expressed as mean ± SEM from 4 independent experiments. **(A)** One-way ANOVA; **(B)** Two-way ANOVA: *p < 0.05, genotype effect

We then assessed if under OXPHOS-dependent conditions, which mimic the metabolic challenges faced by vulnerable organs in m.8993T > G related disorders [47], MF-094 could decrease mutant mtDNA load. To do this, we performed a 3-weeks treatment of cells harboring 80% of mutant mtDNA load with 1 and 10 µM of MF-094. 10 µM MF-094 reduced the mutant mtDNA load by nearly 5% (Fig. 5A), although without reaching statistical significance (ANOVA *F*_2,9_= 3.095). Under these conditions, MF-094 also did not significantly impact cell proliferation (interaction *F*_2,17_= 1.301; genotype *F*_1,17_= 19.24; treatment *F*_2,17_= 2.476) (Fig. 5B). Altogether, these results suggest that MF-094 did not significantly reduce m.8993T > G mtDNA mutation under either glycolytic or OXPHOS-dependent conditions.

## Discussion

Mutations in mtDNA accumulate with age and contribute to mitochondrial dysfunction. Unlike nuclear DNA mutations, mtDNA mutations are the most common cause of primary mitochondrial disease in adulthood [1, 2]. Given the absence of effective treatment options for mtDNA diseases [4] and the strong correlation between clinical severity and the levels of mutated mtDNA [2], we tested a pharmacological strategy aimed at reducing the mutant mtDNA load in a cell model of a heteroplasmic mtDNA mutation.

We used a model with the m.8993T > G point mutation, a relatively common mtDNA mutation affecting the ATP6 subunit of mitochondrial ATP synthase [3]. The ATP synthase functions as a turbine that harnesses the proton flux from respiration to synthetise ATP, thereby regulating mitochondrial membrane potential and cellular energy status. The m.8993T > G mutation causes the substitution of a leucine, a non-polar amino acid, with arginine, a highly polar, cationic amino acid. This electrostatic alteration could hinder proton translocation through ATP synthase and affect the coupling efficiency between proton transport and ATP synthesis [48]. The malfunctioning of ATP synthase and the reduced ATP levels can secondarily impact the respiratory chain, being associated with reduced levels of several respiratory chain subunits [34] and defective respiratory chain complex assembly [47] in cells harboring the m.8993T > G mutation. This mutation is the most common cause of Maternally Inherited Leigh Syndrome (MILS), a fatal encephalomyelopathy in childhood, and Neuropathy, Ataxia, Retinitis Pigmentosa syndrome (NARP) in adults [47]. Previous studies have used cell models with the m.899T > G mutation [34, 49, 50]; however, due to potential variability in mutant mtDNA loads and phenotypes [51, 52], we started by confirming the genotype and phenotype of the cellular model.

We found bioenergetic alterations resulting from reduced mitochondrial activity and a compensatory dependence on glycolysis, evidenced by lower oxygen consumption and higher extracellular acidification of mutant cells compared to wild-type cells. Glycolysis appears to meet basal energy demands, enabling mutant cells to proliferate similarly to wild-type cells in glycolytic medium. This compensatory mechanism aligns with reports showing that tissue ATP levels in several patients with OXPHOS deficiency are similar to those in healthy controls [53]. Activation of compensatory pathways, such as glycolysis, was further supported by resazurin metabolism results in the presence of mitochondrial inhibitors: mutant cells maintained or even increased resazurin metabolism, whereas wild-type cells showed reduced metabolism when challenged with classical mitochondrial inhibitors in the presence of glucose. The mitochondrial inactivity found in mutant cells cultured in glycolytic conditions correlates with an elevated NADH/NAD^+^ or NADPH/NADP^+^ ratio [45], which could explain the increased reduction of resazurin to resorufin in mutant cells challenged with the highest concentrations of the mitochondrial inhibitors antimycin and oligomycin. Consistently, mutant cells were metabolically impaired in the absence of glucose, showing slower proliferation and reduced ATP levels in OXPHOS-dependent conditions.

Induction of mitophagy has shown beneficial effects in several pathologies, including promoting a healthy aging [20, 54]. Stalled mitophagy may contribute to the accumulation of dysfunctional mitochondria in several disorders [15], including the accumulation of mtDNA mutations. Studies in *C. elegans* have shown that tissues prone to accumulate mtDNA mutations have lower mitophagy levels [55]. Accordingly, a very recent study in cells with high m.5024 C > T mutation load showed reduced mitophagy compared to control cells [33]. Here, we also show that cells harboring 80% of mutant mtDNA load presented lower levels of mitophagy than wild-type cells, suggesting impaired mitophagy process or impaired targeting of defective mitochondria for degradation. To investigate the latter hypothesis, we studied the inhibition of the mitochondrial deubiquitinase USP30 as a strategy to target mitochondria for degradation and to induce mitophagy. USP30 inhibition has been reported to selectively enhance basal mitophagy and pexophagy (due to USP30 presence in peroxisomes), without affecting general autophagy [22, 56]. Additionally, the safety of USP30 inhibition is supported by the viability and lack of overt pathology in USP30 knockout mice [26].

We used MF-094, a naphthylsulfonamide, to inhibit USP30 [46]. MF-094 is a potent and selective USP30 inhibitor, exhibiting an IC_50_ of 0.12 µM for USP30, while displaying only 30% inhibitory activity against a panel of 22 isolated ubiquitin specific proteases up to 10 µM [46]. Although the precise mechanism of action of MF-094 has not been fully characterized, it was shown that benzosulfonamides prevent ubiquitin from binding to the active site of USP30, decreasing its deubiquitinase activity [57]. MF-094 has been investigated in other disease contexts: it improved neurological recovery and reduced inflammatory processes response in mice following subarachnoid hemorrhage [31] and promoted wound healing in diabetic rats [58].

Here, we show that MF-094 increased mitochondrial protein ubiquitination, including TOM20 ubiquitination within the first 2 h of treatment, and mitolysosome formation, which was detected after 24 h of treatment. TOM complexes are known targets of USP30; however, it has been reported that USP30 associates with only a small fraction of TOM complexes [23]. This may explain the relatively small proportion of ubiquitinated vs. non-ubiquitinated TOM20 in the presence of the USP30 inhibitor. Moreover, sustained ubiquitination of other proteins such as mitofusin 2 by USP30 inhibition has also been linked to mitophagy [31]. Mechanistically, USP30 acts upstream of the PINK1 kinase, setting a threshold for mitophagy initiation [59, 60]. PINK1 phosphorylates ubiquitinated proteins on the mitochondrial surface, recruiting the E3 ubiquitin ligase Parkin [61]. USP30 inhibition sustains ubiquitination of OMM proteins, increasing the bioavailability of ubiquitinated substrates for PINK1 [59, 60]. The observed increase in mitolysosome formation with MF-094 treatment – detected by an increased number of mitochondria in an acidified environment – suggests no defect in the recruitment of mutated mitochondria into the autophagosome. However, we cannot exclude the possibility of a defect in lysosomal degradation that could hinder the clearance of mutated mtDNA.

Dysfunctional ATP synthase, as caused by the m.8993T > G mutation, can disrupt respiratory chain function, leading to altered mitochondrial membrane potential and energy production [62]. In turn, the resulting bioenergetic impairment can reduce mitochondrial membrane import of proteins [63]. Both reduced mitochondrial membrane potential and mitochondrial membrane import favor PINK1 accumulation on the mitochondrial surface [64]. Since USP30 acts upstream of PINK1 [59, 60] and mitophagy plays a crucial role in the turnover of mitochondrial nucleioids as previously observed in neurons [65], we hypothesized that USP30 inhibition might promote the clearance of mitochondria with higher mutant mtDNA loads, allowing selective biogenesis of healthier mitochondria. Consistent with this hypothesis, a very recently published work showed a ~ 10% reduction in m.5024 C > T mtDNA mutation load in the offspring of mothers with USP30^−/−^ genotypes, as well as a 3–5% reduction in m.5024 C > T mtDNA mutation load in cells cultured under OXPHOS-dependent conditions following pharmacological USP30 inhibition with CMPD39 [33].

We found a spontaneous decrease in mutant mtDNA load over time in culture in cells initially harboring a 40% mutant mtDNA load, consistent with heteroplasmic shifting in dividing cells favoring healthier cells [51]. MF-094 did not significantly affect this parameter in cells containing either 40 or 80% mutant mtDNA load under glycolytic conditions. Under OXPHOS-dependent conditions, MF-094 reduced the mutant mtDNA load by nearly 5%, although without reaching statistical significance. It is however uncertain if using other experimental approaches that yield more data, such as single-cell heteroplasmy analysis [33], could identify a statistical significance. Additionally, the reduced proliferation of cells treated with MF-094 may be related to increased apoptosis, which can hinder the reduction of mutant mtDNA load. Indeed, USP30 regulates apoptosis and its inhibition has been shown to sensitize cells to death [66].

The maintenance (or non-significant reduction) of mutant mtDNA load in our experiments after MF-094 treatment suggests that targeting the threshold for mitophagy initiation may be insufficient to reduce mutant mtDNA load in this context of m.8993T > G related diseases (NARP/MILS). Follow-up studies are needed to clarify whether the mitophagy induced by MF-094 is sustained over the chronic treatment or whether mitochondrial biogenesis effectively replaces degraded mitochondria. If mitophagy induced by USP30 inhibition is not sustained, strategies that favor mitophagy following ubiquitination, such as promoting PINK1 activity, could complement USP30 inhibition to increase the mitophagy of dysfunctional mitochondria [64, 67]. Alternatively, promoting mitochondrial fission may facilitate mitophagy of dysfunctional mitochondria by generating smaller mitochondria more suitable for autophagosome engulfment [68]. However, few pharmacological strategies have been successfully developed to promote mitochondrial fission or selectively increase PINK1 activity [69, 70]. If mitochondrial biogenesis is insufficient to replace degraded mitochondria, several pharmacological approaches warrant investigation, primarily those that favor the activity of the transcription factor PGC-1α, such as activators of the AMPK pathway – an essential metabolic regulatory pathway activated by energy deprivation [11].

In summary, this study addresses the underexplored question of whether pharmacological inhibition of the mitochondrial deubiquitinase USP30 is a potential therapeutic strategy in the context of mtDNA diseases. While our findings indicate that pharmacological USP30 inhibition with MF-094 induces mitophagy, a significant decrease in the mutant mtDNA load was not achieved, suggesting that MF-094 has little potential to manage m.8993T > G-related mtDNA diseases such as NARP and MILS. The expansion of this study using other USP30 inhibition strategies, whether genetic or pharmacological, could strengthen our conclusions about the therapeutic potential of USP30 inhibition in m.8993T > G-related mtDNA diseases. Further studies on different mtDNA mutations are needed to understand if the efficacy of USP30 inhibition is mutation-dependent. This study represents an effort to develop a mitochondria-targeted strategy to induce mitophagy in mtDNA diseases, and highlights the complexity of mitophagy pathways and the intricate regulation of mtDNA dynamics in this context.

## Supplementary Information

Below is the link to the electronic supplementary material.

Supplementary Material 1

## Data Availability

The data that support the findings of this study are available from the corresponding author upon reasonable request.
