# Supplementary material for: Targeting mitochondrial deubiquitinase USP30 to induce mitophagy in heteroplasmic mitochondrial diseases
Source: Pharmacol Rep. 2026 Jan 26;78(2):519–34. doi: 10.1007/s43440-026-00829-7 (PMC12975861; doi:10.1007/s43440-026-00829-7)
Supplement: Supplementary file 1 — Supplementary Material 1 [file 43440_2026_829_MOESM1_ESM.pdf]

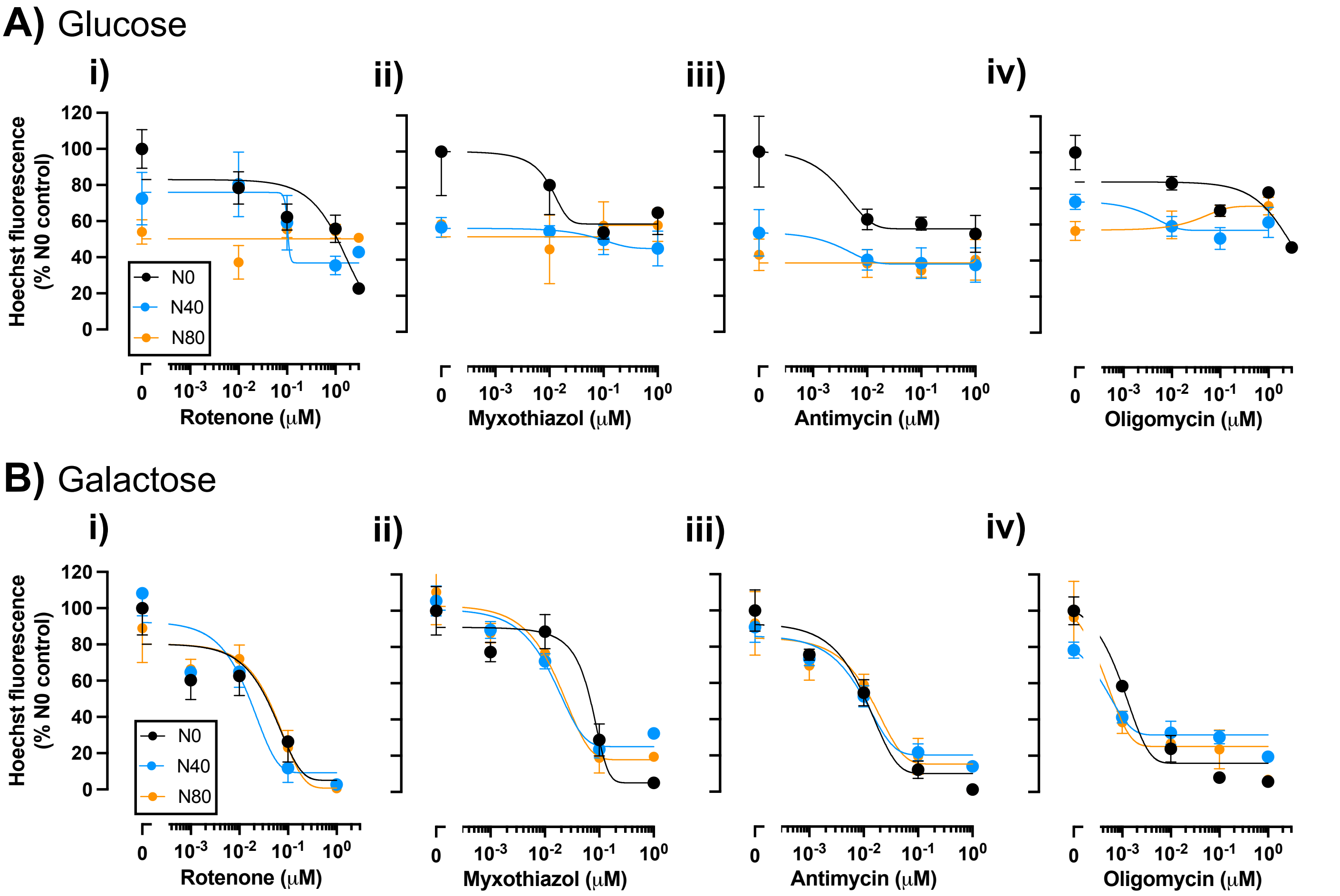

Supplementary Figure 1. Hoechst fluorescence, indicative of cell density, was measured after the treatment of 143B cybrid cells harboring 0% (N0), 40% (N40) or 80% (N80) of the m.8993T>G mtDNA mutation cultured in **A)** glucose- and **B)** galactose-containing medium with increasing concentrations of classical mitochondrial inhibitors for 24 h: **i)** rotenone (complex I inhibitor); **ii)** myxothiazol (complex III inhibitor); **iii)** antimycin (complex III inhibitor); **iv)** oligomycin (ATP synthase inhibitor). Data are mean ± SEM in % of N0 without drug treatment; n = 4 independent experiments.

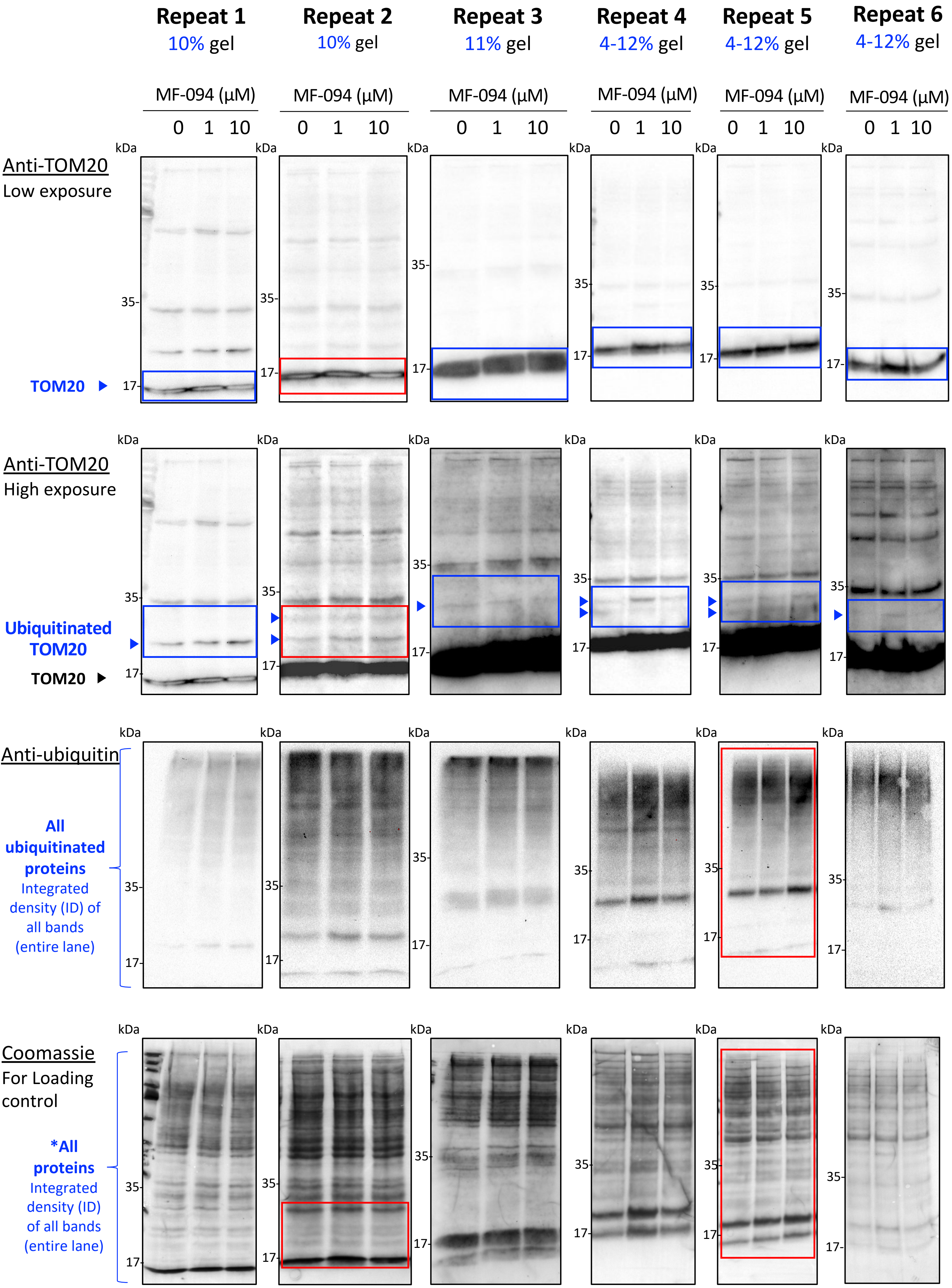

Suplementary Figure 2. Original western blots of 6 independent experiments. Red boxes show the cropped western blots present in the Figure 3C and 3D. Note that gels have different polyacrylamide %, explaining small variations in protein migration.
